# Presentation to Dr. Hunt

**Published:** 1862-09

**Authors:** 


Presentation to Dr. Hunt.—Dr. S. B. Hunt's friends, medical, editorial and personal, have presented to him, since his departure from the City, a sword, belt, sash and pistol, as a memento of their appreciation of his patriotic devotion to his country, and distinguished ability to discharge the duties of the appointment he has accepted.

